# Agriculture: Tracking Antibiotics in Groundwater

**Published:** 2004-09

**Authors:** Julia R. Barrett

Antibiotics are commonly used in food animal production to treat illness, promote growth, and ward off disease. These drugs and their metabolites appear in animal wastes and can eventually enter ground and surface waters following the common practice of applying manure to agricultural fields. Given that low levels of antibiotics can promote the development of microbial drug resistance, their presence in ground and surface waters constitutes an environmental health concern. Current methods for measuring trace amounts of antibiotics in water samples are costly and time-consuming, but researchers now show that a common food-test kit yields comparable information quickly and cheaply.

Researchers led by Kuldip Kumar at the University of Minnesota describe in the January–February 2004 *Journal of Environmental Quality* their use of the kits, which rely on the enzyme-linked immunosorbent assay (ELISA), a widely used technique based upon antibody recognition of target compounds. Food inspectors use the kits to test for drug residues in meat and milk. Using the kits, the researchers found trace amounts of tylosin, tetracycline, and chlortetracycline in surface and ground waters, field runoff, and swine manure. These results were confirmed with liquid chromatography–mass spectrometry (LC-MS). “Our bigger [question] is whether this small concentration of antibiotics in the environment is producing antibiotic-resistant bugs,” says Kumar.

The researchers, who are among the first to employ ELISA to test environmental samples for antibiotics, say the assay is as sensitive as LC-MS for detecting target compounds in parts per billion, but is quicker, easier, and less expensive ($5–15 per sample, compared to about $150 for LC-MS, including sample preparation and instrumentation). However, ELISA would best serve as a screening tool rather than a means of precise quantitation, because structural similarities between antibiotics, their metabolites or degradation products, and other compounds can yield false-positive results due to cross-reactivity. For example, the tetracycline test used by the researchers detected not just that drug but also several others in the same class.

Chemist Diana Aga of the University at Buffalo, who has also used ELISA to detect antibiotics in environmental samples, concurs that cross-reactivity is its key limitation. “This method shouldn’t be the basis of any policy making because ELISA is a semi-quantitative technique,” she says. “It’s a good technique because it is cheap and easy and fast, but it could also give you some false-positives or overestimate results.”

Despite this limitation, ELISA is a useful tool, says Ching-Hua Huang, an environmental engineer at the Georgia Institute of Technology. Researchers might use it to rapidly evaluate the presence of antibiotics in the environment, identify hot spots, and use the information for further studies. There is little dispute that antibiotics are in our source waters, says Huang—the question now is whether, and how, these compounds are linked to adverse effects in the environment.

## Figures and Tables

**Figure:**
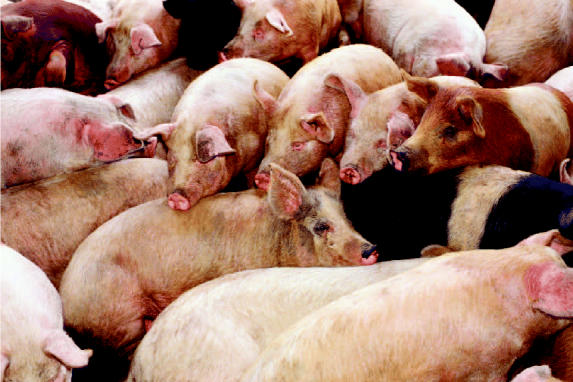
**Farm folly?** Animal antibiotic use may contribute to microbial resistance, making it important to track these drugs in the environment.

